# vexptoolbox: A software toolbox for human behavior studies using the Vizard virtual reality platform

**DOI:** 10.3758/s13428-022-01831-6

**Published:** 2022-03-23

**Authors:** Immo Schuetz, Harun Karimpur, Katja Fiehler

**Affiliations:** 1 Experimental Psychology, Justus Liebig University, Otto-Behaghel-Str. 10 F, 35394 Giessen, Germany; 2 Center for Mind, Brain and Behavior (CMBB), University of Marburg and Justus Liebig University Giessen, Giessen, Germany

**Keywords:** Virtual reality, Virtual environments, Behavioral study, Experiment development, Vizard programming

## Abstract

Virtual reality (VR) is a powerful tool for researchers due to its potential to study dynamic human behavior in highly naturalistic environments while retaining full control over the presented stimuli. Due to advancements in consumer hardware, VR devices are now very affordable and have also started to include technologies such as eye tracking, further extending potential research applications. Rendering engines such as Unity, Unreal, or Vizard now enable researchers to easily create complex VR environments. However, implementing the experimental design can still pose a challenge, and these packages do not provide out-of-the-box support for trial-based behavioral experiments. Here, we present a Python toolbox, designed to facilitate common tasks when developing experiments using the Vizard VR platform. It includes functionality for common tasks like creating, randomizing, and presenting trial-based experimental designs or saving results to standardized file formats. Moreover, the toolbox greatly simplifies continuous recording of eye and body movements using any hardware supported in Vizard. We further implement and describe a simple goal-directed reaching task in VR and show sample data recorded from five volunteers. The toolbox, example code, and data are all available on GitHub under an open-source license. We hope that our toolbox can simplify VR experiment development, reduce code duplication, and aid reproducibility and open-science efforts.

## Introduction

In traditional lab-based behavioral studies, participants typically sit motionless in front of a screen while viewing stimuli that are highly controlled in position and timing. Participant behavior is often measured using stereotypical responses such as goal-directed reaching movements or simple button presses. In contrast, virtual (VR) and mixed reality (MR) setups can create a much closer approximation of the real world and allow researchers to study dynamic human behavior (often described as an “active observer”, e.g., Wexler & van Boxtel, 2005) in highly naturalistic environments (Clay et al., 2019; Fox et al., 2009; Scarfe & Glennerster, 2015; Wexler & van Boxtel, 2005). VR and MR setups further allow to track the observer’s movement in space and to present real-time visual and auditory input that closely matches the spatial layout expected from a real environment (Scarfe & Glennerster, 2019; Slater, 2009; Troje, 2019). At the same time, the experimenter retains full stimulus control and reproducibility (Fox et al., 2009), rendering VR and MR powerful research tools in themselves even when not striving for a faithful reproduction of reality (de Gelder et al., 2018). Over the past decade, the use of VR and MR for human behavior research has steadily grown in popularity, aided by the increasing availability of high fidelity, affordable consumer hardware (Fox et al., 2009; Pan & Hamilton, 2018; Slater, 2018; Troje, 2019). In the past, optimal use of these technologies often required professional software development skills. However, the accessibility of 3D rendering platforms has made great strides with the rise of consumer VR, and research-specific toolkits to aid in experimental design and analysis have recently started to gain momentum. Here we present vexptoolbox, a novel open-source toolbox that facilitates the implementation of behavioral VR experiments using the Vizard platform.

Various software packages to aid in designing and implementing a virtual environment (VE) are now readily available to researchers. The most commonly used include Unity (Unity Technologies, San Francisco, CA, USA) and Unreal Engine (Epic Games, Cary, NC, USA), originally created as game engines and now used in countless games and interactive experiences (both 2D and 3D), as well as Vizard (WorldViz, Santa Barbara, CA, USA), a development environment and rendering engine specifically designed for academic and industry research and development. Unity and Unreal are free to use for non-commercial research purposes, while Vizard requires a commercial license but includes a time-limited free license option. All platforms require a certain amount of software development expertise to use to their full potential: Vizard is based on the Python programming language, while Unity and Unreal are programmed (“scripted”) in C# and C++ , respectively. To enable researchers to easily build complex environments, all software packages also offer a built-in 3D scene editor and provide a number of geometric shapes and freely usable 3D objects (“assets”) that can be used when arranging a virtual scene. With careful selection of the right software and hardware components, almost any experimental scenario can now be implemented as a virtual environment.

In contrast to the creation of the virtual scene, the implementation of experimental designs and data collection code still poses a challenge to researchers. None of the cited renderers provide capabilities out of the box to solve typical research study tasks, such as controlled stimulus presentation and precise recording of time-stamped behavioral data (i.e., discrete button press responses or continuous body or eye movements). Behavioral studies are typically structured around a collection of individual stimulus–response presentations or *trials*. Individual trials are often generated based on one or multiple factors that are systematically varied by the experimenter (*Independent Variables* or *IVs*), and in each trial one or more behavioral outcomes are measured (termed *Dependent Variables* or *DVs*). Trials are usually presented in pseudo-random order to reduce or eliminate effects based on the order of specific manipulations, and multiple repetitions of individual trials may be used to achieve a better estimate of a noisy behavioral outcome. To give an example: If participants are asked to perform repeated reaching movements to a visual target in VR using a handheld controller, the position of the target could be manipulated systematically as an IV, the controller’s movement trajectory could be considered a DV, and individual movements would form individual trials. While this type of structure is easy to implement with some programming experience, standardized frameworks for behavioral experiments can help researchers avoid “reinventing the wheel” by providing code for common tasks, and boost reproducibility and open science efforts by making it easier to share and compare experiment code.

In addition to discrete (per-trial) measures such as reaction times or button press responses, VR allows to continuously track and record participant behavior while interacting with the environment. Examples of continuous behavioral measures include body posture, head and hand kinematics, and dynamic eye movement behavior. All current VR systems track the observer’s head using sensors on the head-mounted display (HMD) to allow for perspective-correct stereoscopic rendering, and often also provide hand position and even finger tracking through the use of hand-held controllers, often at millimeter-scale resolution (Bauer et al., 2021; Shum et al., 2019; but see also Bauer et al., 2021; Niehorster et al., 2017; Peer et al., 2018 for systematic errors that can arise with these systems). Some labs further utilize professional motion tracking solutions like OptiTrack (NaturalPoint, Inc., Corvallis, OR, USA) or VICON (Vicon Motion Systems Ltd, Oxford, UK), which can capture and record full-body motion data. Body kinematics are useful to study, e.g., human spatial perception and navigation abilities (Karimpur et al., 2020; Klinghammer et al., 2016; Pastel et al., 2021), and full-body animated avatars are an important tool in research areas such as presence (e.g., Slater & Steed, 2000; Slater & Usoh, 1993) and embodiment (e.g., Kilteni et al., 2012; Pan & Steed, 2019). Another type of continuous behavior of interest to researchers is eye movement data (Clay et al., 2019), which is becoming more widely accessible through increasingly available eye tracking solutions for VR, such as in the FOVE (FOVE Ltd., Torrance, CA, USA) or HTC Vive Pro Eye HMDs (HTC Corp., Xindian, New Taipei, Taiwan). VR eye tracking allows to capture an observer’s gaze behavior during exploration of highly realistic and complex environments (Clay et al., 2019; Hayhoe & Rothkopf, 2011; Rothkopf et al., 2007), visual search (Helbing et al., 2020; Kit et al., 2014; Marek & Pollmann, 2020) or visual working memory tasks in VR (Draschkow et al., 2021, 2022), or to directly compare the deployment of gaze while walking in a real building versus navigating its virtual twin (Drewes et al., 2021). Other continuous measures might come from mobile physiological sensors or EEG devices (e.g., Banaei et al., 2017; Gramann et al., 2014). None of the rendering engines listed above provide recording facilities for this type of continuous data out of the box. Therefore, an experimenter is often left to write their own background routine to sample the desired data (for example, whenever the HMD display is refreshed), perform additional processing or filtering, and then stream the data to disk or record it in memory until the end of a trial. Providing this functionality in the form of a programming toolkit or framework can not only speed up the implementation of a given experiment, but could also provide a common, documented format for continuous data across different labs and projects.

Experiment frameworks for traditional screen-based paradigms have been available for decades, although they differ in goals and functionality. Written in MATLAB and often considered the gold standard for visual psychophysics, the Psychophysics Toolbox (Brainard, 1997; Kleiner et al., 2007) is aimed at easy creation and highly accurate presentation of visual and auditory stimuli, but also includes functionality for common experimental tasks such as response recording. PsychoPy (Peirce, 2007) and OpenSesame (Mathôt et al., 2012) are both written in the Python programming language and aim to be complete experimentation platforms that provide functionality for experiment design, stimulus delivery and data acquisition through a growing library of backends and plugins. Finally, commercial solutions to experiment frameworks exist as well, such as Presentation (Neurobehavioral Systems, Inc., Berkeley, CA, USA) or E-Prime (Psychology Software Tools, Inc., Pittsburgh, PA, USA). An extensive review of currently available frameworks with a focus on timing accuracy was recently published by Bridges et al. (2020). Only a few of these packages currently have some support for VR at all (Psychtoolbox and PsychoPy at the time of writing), but various frameworks to facilitate the implementation of VR behavioral studies on top of the major rendering engines have recently emerged as well. Some of these packages do not aim to be a comprehensive solution for building experiments, but are instead designed as add-ons to solve specific recurring tasks. An example is the Toggle Toolkit (Ugwitz et al., 2021) to quickly implement scene changes based on user behavior. Others are designed as full experiment frameworks geared towards a specific type of paradigm or research question, such as the Landmarks toolkit for spatial navigation experiments (Starrett et al., 2020). Finally, a number of recent packages aim to provide a general purpose framework for behavioral experiments using the Unity engine. The Unity experiment framework (UXF; Brookes et al., 2019) contains an extensive set of C# classes for trial-based experiment flow control and behavioral data collection. It supports both VR and traditional screen-based scenarios, including in a web browser. Providing similar functionality, bmlTUX (Bebko & Troje, 2020) also includes a graphical user interface (GUI) to set up complex factorial experimental designs and was specifically built for easy integration with VR experiments.

While a growing number of packages for behavioral experimentation are now available for the Unity platform, no such toolkits exist for WorldViz’s Vizard environment at the time of writing. At the same time, Vizard is being used in our and many other behavioral research labs around the world (WorldViz Inc., 2020), and its use of Python as scripting language makes it particularly accessible due to Python’s pervasiveness and focus on being easy to learn. Inspired by the list of software tools described above, as well as built on experience from creating virtual reality paradigms in our lab at Justus-Liebig University Giessen, we here present a Python toolbox to facilitate the implementation of behavioral VR paradigms using the Vizard platform. Additionally, we are making our code freely available on GitHub under an open-source license and hope that our toolbox can help other researchers in implementing common elements of behavioral experiments, reducing code reuse, and improving study reproducibility.

In the following sections of this manuscript, we will first outline the design decisions behind and the features of our toolbox. We will then describe a simple visuo-motor VR experiment that was built using our software components and can serve as example code for prospective users, followed by summarizing some of the behavioral data and measures from a small-scale (*N* = 5) example data collection performed in our lab. The paradigm and recorded example data are made available as a separate GitHub repository^1^ that provides a starting point to other researchers interested in using our toolbox.

## Experiment toolbox

A main goal of the software described here is to help researchers in developing experimental code while still retaining the full flexibility of Python and the Vizard scripting environment. We aim for the toolbox to be easy to use, which is reflected in some of the design decisions made. First, our code does not depend on external Python libraries beyond those bundled with Vizard, as those may not be available or easily installable on a lab computer with limited network connectivity. Second, it can be quickly added to any project by copying a folder and adding an import statement to the main Python script file. And third, we chose to rely on Vizard’s general interface methods for sensor objects instead of integrating device-specific SDKs, meaning that any hardware that is supported by Vizard can be used by features such as position and orientation recording.

### Installation

The toolbox is available from a public GitHub repository^2^ and licensed under the MIT license. It can be added to a project by cloning or downloading the latest release from GitHub and copying the *vexptoolbox* subfolder into the folder where the user’s main experimental script is stored. Alternatively, direct download and installation is also supported via Python’s *pip* installer and Vizard’s built-in package manager, which can install the toolbox to be globally accessible by all Vizard scripts and centrally updated if necessary. It can then be imported into the experiment script by adding *import vexptoolbox* near the top of the script. Published releases are regularly tested using both Vizard 6 (which is based on Python 2.7) and Vizard 7 (based on Python 3.8 +).

### Trial-based experimental design

**Fig. 1:**
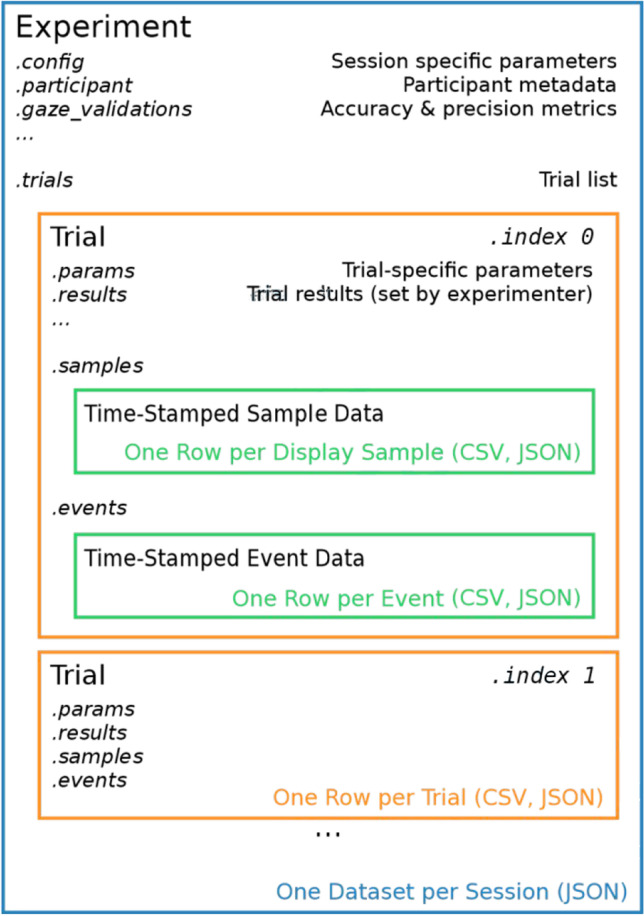
Overview of the Python classes used in vexptoolbox to encapsulate the experimental design. *Labels set in italics* indicate data structures stored as attributes of the corresponding object. *Colored text* describes the general data format (i.e., what is represented by a single row of output data) and supported output file types

A main goal of our toolbox is to provide functionality for common tasks in a behavioral experiment, such as loading or generating an experimental design, looping through trials, collecting trial results, and saving all data in well-defined file formats that can be easily analyzed further. We chose an object-oriented approach to encapsulate the typical components of a behavioral study and thus implemented Python classes for the experiment as well as individual trials. Figure 1 illustrates the high-level structure of these Python objects and the information stored in each class.

A top-level *Experiment* object combines all information that is specific to an entire experimental session. Currently, this includes participant information such as a participant code and demographic data, results from calibration and validation procedures, and a config data structure that can hold parameters that apply to the entire session, such as global stimulus or timing information. Participant metadata can be stored manually or requested from the experimenter via a form built on Vizard’s vizinfo UI functionality. Additionally, the Experiment object holds a Python list of *Trial* objects for the current experiment and offers class methods to add, randomize, start, and stop trials. Trial objects are created by importing a tabular text file of trial parameters, or generated via Python code such as by specifying the number of factors and levels of a full-factorial design. Randomization is also available, either by shuffling all trials before presenting or by shuffling only within ordered experimental blocks using an optional block attribute. The block number is implemented as a property of each trial (rather than a separate loop over experimental blocks, cf. Brookes et al., 2019), and a column of the input file can be used to specify block numbers. Each Trial object contains all data pertaining to a specific experimental trial, most importantly a *params* attribute for trial-specific stimulus parameters that is initialized on trial creation or read from a CSV file, and an initially empty *results* attribute that the experimenter can use to store collected result data for this trial, simply by assigning key-value pairs akin to a Python dictionary. Most of these data structures are implemented using a custom data structure (ParamSet) which can be used identically to a Python dictionary, but which adds functionality such as easy import and export of parameters from CSV and JavaScript object notation (JSON) file formats. In some cases, Trial objects can contain additional data, such as timestamped position and orientation data if the *SampleRecorder* component (see below) is used to record continuous participant behavior. Experiment data can be saved to different output file formats, either individually after a trial is finished or after all trials in the trial list have been run. The entire Experiment structure including all trial data and parameters can be saved as JSON format, which yields a compact, single-file data structure that is ideal for Python-based data analysis. Additionally, trial params and results can be combined into a CSV file containing one row per trial, and continuous sample and event data can be exported to a CSV file in which each line represents a single data sample at display frame rate (cf. Figure 1). Trial result data can be written to disk at the end of an experimental session or as separate files immediately after each trial, which can limit data loss in case of script or data recording errors.

The toolbox provides two main ways for experimenters to run the individual trials in order. First, they can write a Python loop, iterating over the *Experiment.trials* argument and calling the corresponding functions to start and end each trial directly. This method is well suited for relatively simple experiments and when converting existing experimental code to vexptoolbox. Second, they can use the built-in *run()* method and provide at least one task function^3^ that will be run for each trial while being passed the corresponding Trial object for easy access to parameters and result data. Currently, trials can be split into a main as well as a pre- and post-trial task function, which are automatically called in the correct order. Pre- and post-trial tasks can for example be used to set up specific stimulus properties or communicate with external hardware such as EEG amplifiers without cluttering the main trial implementation. Our example study described below includes examples of a main and a pre-trial task.

### Recording continuous behavior

**Fig. 2:**
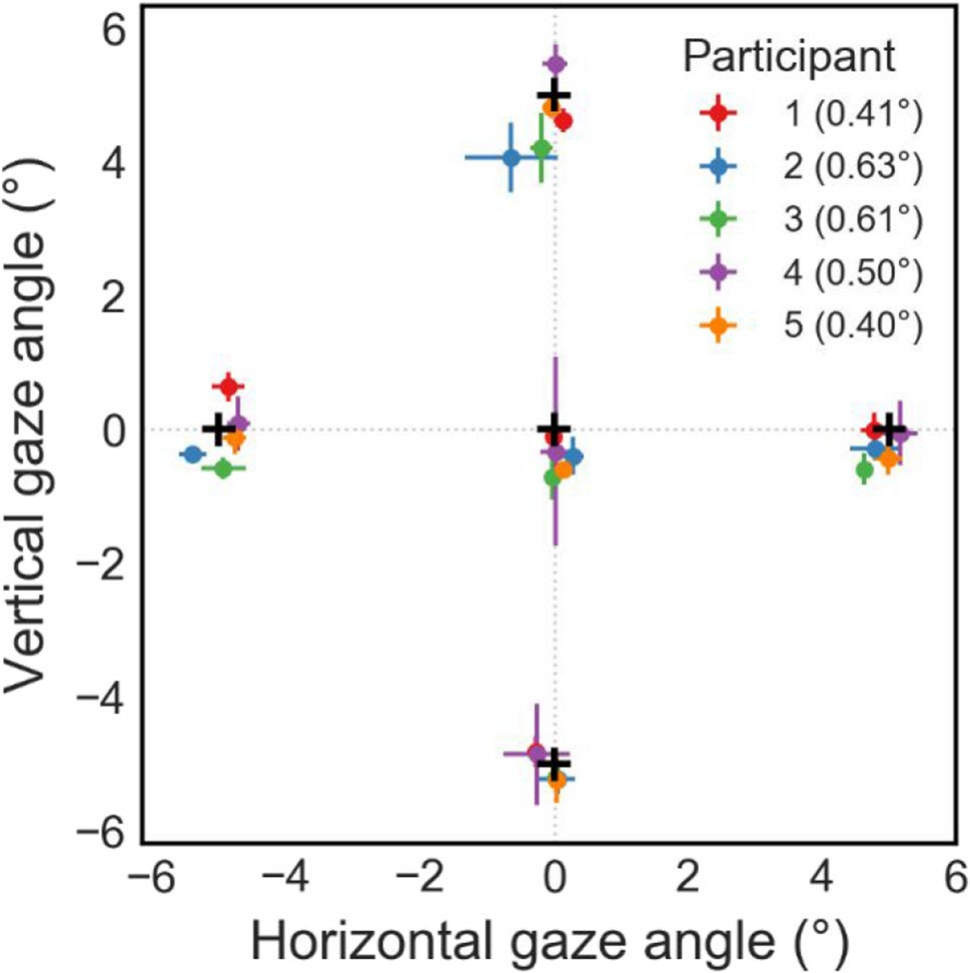
Eye-tracking accuracy measured using our validation procedure in the example experiment. *Black crosses* indicate target positions (central and ± 5° relative to the point in between the participant’s eyes). *Colored markers* indicate average angular gaze position for each target and participant, error bars the standard deviation of gaze samples during fixation. *Numbers in parentheses* denote each participant’s average gaze accuracy across all five targets

Besides storing discrete result data like a “yes” or “no” response, which usually occurs once per trial, our toolbox also includes a *SampleRecorder* class that can record continuous behavioral measures such as movement in the environment over the course of a trial. A major use for this type of recording is to collect participants’ eye movement behavior. Various VR and MR HMDs now include eye tracking, and gaze placement in a virtual environment can be informative about a participant’s cognitive and perceptual processes (Clay et al., 2019). If a Vizard-compatible eye tracker is specified when the SampleRecorder component is instantiated, gaze vectors output by the eye tracker are automatically included in the recorded data. Eye-tracking data requires further processing compared to e.g., controller movement data, because the gaze vectors are usually reported relative to the tracking device’s frame of reference and need to be converted to world space coordinates. Therefore, the recorder component automatically computes and logs the gaze origin and direction vector in world space. Additionally, an intersection test of the resulting world gaze vectors with the scene is performed automatically on each frame (often referred to as raycasting). If the current gaze direction intersects with an object, the coordinates of the closest point of intersection together with the identity of the object hit by the gaze ray are logged as well. The most recent gaze direction, position, and fixated object are also made accessible as attributes of the SampleRecorder object, which enables the experimenter to easily detect participant fixations on different scene elements or confirm that an observer is holding their gaze where they were instructed to fixate. Finally, the spatial accuracy and precision of an eye tracker are important measures in determining how well different fixated objects can be distinguished (Holmqvist et al., 2012), but not all eye tracker manufacturers report real-world performance metrics in addition to their technical specifications. Additionally, for some devices such as the Vive Pro Eye, the calibration routine available in Vizard only reports success or failure, but does not report metrics of data quality. To be able to assess the eye tracker’s spatial accuracy and precision in a session (i.e., how well a given participant was calibrated), the sample recorder component also includes code to display visual targets at predefined positions and record gaze data while the participant is instructed to fixate on each “validation” target. Data quality metrics such as absolute error, standard deviation (SD) and root mean square error of gaze position are then computed from the deviation between the actual gaze vector and the true target position, a standard approach that has recently been implemented as a Unity package as well (Adhanom et al., 2020). An example of eye-tracking accuracy data for targets at ± 5° from our example experiment is shown in Fig. 2.

### SteamVR debug overlay

Some VR experiments can be run anywhere a SteamVR capable computer and HMD can be set up, including on participants’ own VR setups if distributed appropriately. However, many paradigms require the precise measurement and alignment of the virtual environment with the real-world lab space, for example when displaying a virtual table in the same place as a physical one to provide passive haptic feedback. Additionally, the SteamVR coordinate system tends to change especially after a brief loss of tracking (Niehorster et al., 2017), the indexing of tracked devices such as controllers or Vive trackers can depend on the order in which they are paired and/or powered on, and the local coordinate system of Vive trackers can change depending on their configured “tracker role”. To be able to quickly visualize the current state of the SteamVR system and take measurements, we created a *SteamVRDebugOverlay* component, which can be added directly to an existing Experiment object or used as a standalone component in any Vizard script. The overlay is hidden by default and its visibility can be toggled using a hotkey (F12 by default).

**Fig. 3:**
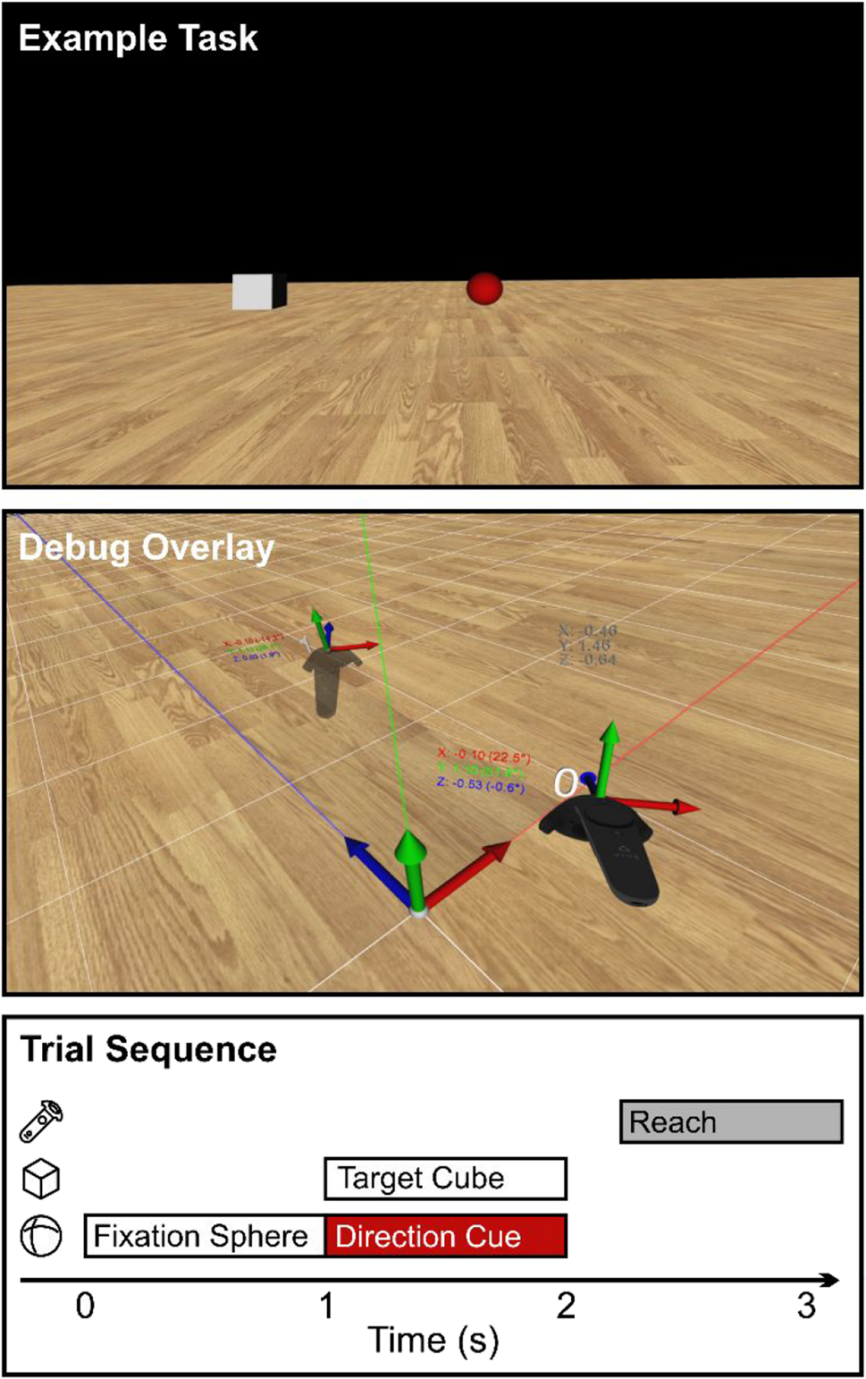
Screen captures of our example VR environment and illustration of the trial sequence. *Top*: Screenshot from the case study pro-/anti-reach task described in Sect. 3. The displayed stimulus arrangement instructs the participant to reach opposite (*red fixation sphere* = anti-reach) of the presented target position (*gray cube*). *Middle*: Example scene with the SteamVR debug overlay enabled. Coordinate axes, alignment lines, and white grid visualize Vizard’s world coordinate system. *Dark gray numbers* indicate HMD position (shown as heads-up display to the user). Two Vive controllers are shown with their index in Vizard’s controller list, current position and Euler angle data, and axes denoting their local coordinate system. *Bottom*: Trial sequence in the example paradigm, corresponding to the scene shown in the top panel. A white fixation sphere (*sphere icon*) is shown for 1 s, then changes color to red to indicate an anti-reach. Simultaneously with the color change, a target cube (*cube icon*) is displayed for 1 s. After both cube and sphere disappear, the participant performs a reach movement with the controller (*gray*; controller icon). Icons by Ben Davis from NounProject.com

All overlay components are created using Vizard built-in object primitives. The debug overlay consists of a) a depiction of the global coordinate system by means of coordinate axes, colored lines, and a grid aligned with the global origin, b) a model representation of each connected SteamVR device (controllers, trackers, base stations) together with its device index, local coordinate system, and current position and orientation data, and c) a UI panel displaying position and orientation data of all nodes tracked by the debugger. An example scene with the overlay enabled is shown in Fig. 3 (middle panel). Other nodes or sensor objects, such as from an external motion tracking system, can be added and visualized as part of the overlay as well. Finally, position and orientation measurements can be marked in 3D space using a controller, and the list of stored measurements as well as the full overlay scene can be exported to a file. These resources can be helpful during experiment development and aid in the design of virtual environments aligned with a real-world lab space.

### Utility functions

Finally, based on frequently reimplemented functionality in our lab, the experiment toolbox also includes a growing list of utility classes and functions. One example is the *ObjectCollection* class, which groups 3D objects (Vizard nodes) in a way similar to the “tag” feature of Unity: An object collection allows to easily show, hide, and change properties of one or multiple objects, which previously had to be implemented by iterating over a list of node objects. Each object’s visibility, position, orientation, and scale can also be manipulated using a single line of code, keeping stimulus presentation code more compact and readable, which is especially useful in paradigms that present different combinations of task-relevant objects (e.g., the breakfast table items used as reach targets in Klinghammer et al., 2016). Other utility functionality includes the aforementioned ParamSet class, which extends a Python dictionary by file export and import features as well as object.attribute notation in addition to dictionary access, and a collection of functions to e.g., present instruction or feedback text to the participant within the virtual environment.

## Example experiment: Pro-/anti-reach task

To illustrate some of the features of our toolbox in more detail, such as the trial-based programming structure, gaze and controller motion recording, and storing of trial result data, we implemented a simple VR experiment using Vizard 6.3, SteamVR, and the current version of vexptoolbox at the time of writing (version 0.1.1). The Python code for this experiment and the analysis described below, as well as example data recorded from volunteers in our lab, are available as a separate GitHub repository, which can serve as an introduction or template for researchers interested in implementing their own paradigm (https://github.com/ischtz/proantireach-vizard). Additionally, the code available on GitHub runs on any SteamVR-compatible computer and HMD with no modifications required, making this also a good demonstration task for students interested in learning experiment programming with Python and Vizard.

### Experimental paradigm

We implemented a pro-/anti-reach task in VR. In this paradigm, participants perform manual reaching movements towards a visually presented target (pro-reach) or to a location in the visual hemifield opposite to the visual target (anti-reach). A similar paradigm was first described for eye movements (pro- / anti-saccade task; Medendorp et al., 2005; Muñoz & Everling, 2004). By independently manipulating visual target information and the motor action required for a correct response, spatial processing in visual and motor areas of the brain can be disentangled. This approach has previously been used to investigate the spatial coding of both visual target and motor goal using methods such as fMRI (Gertz & Fiehler, 2015; Gertz et al., 2017), MEG (Blohm et al., 2019), or primate electrophysiology (Westendorff et al., 2010). We chose this task here for its relatively simple experimental design and because it is well suited to demonstrate the eye- and controller motion-tracking components of our framework.

**Fig. 4:**
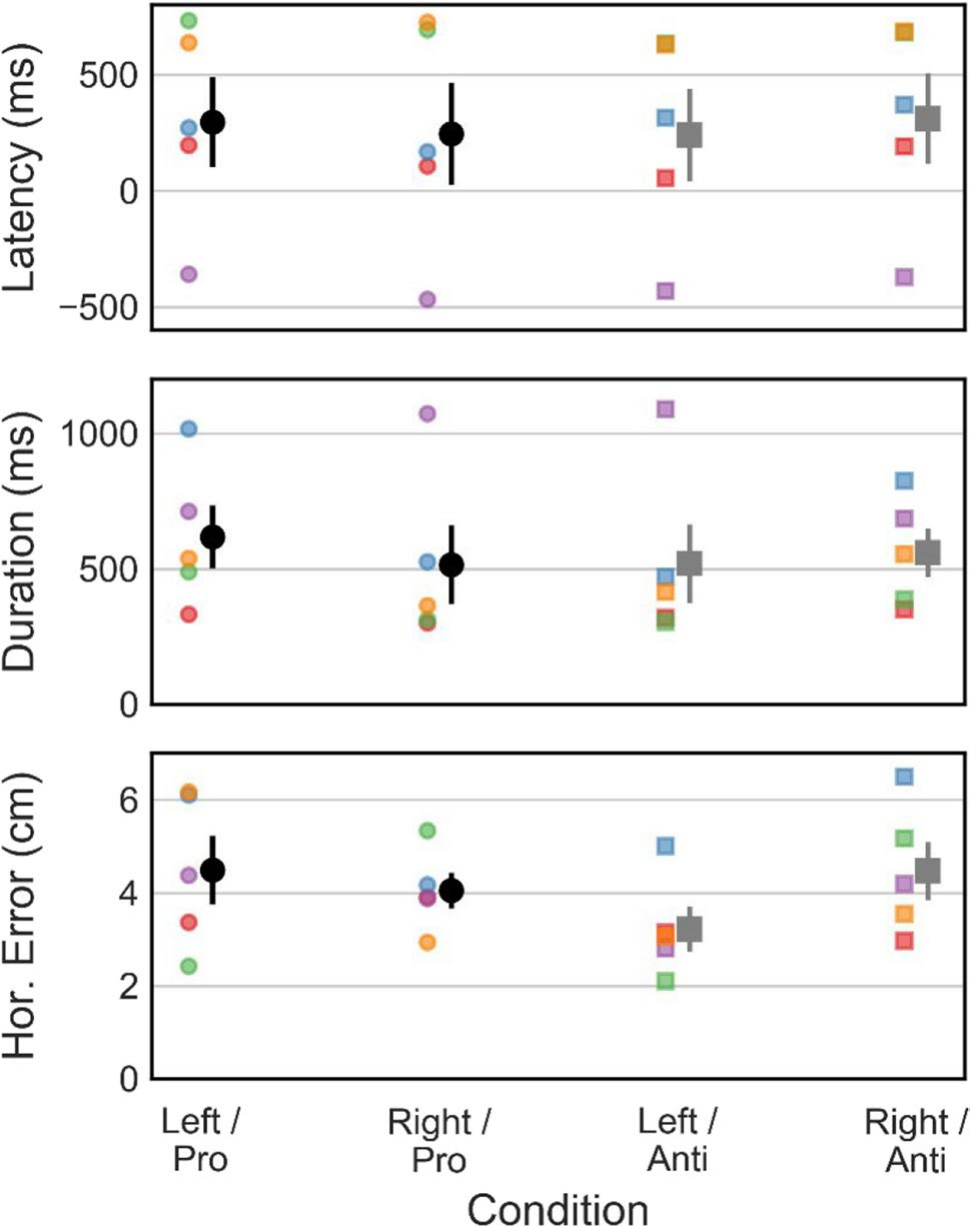
Example behavioral parameters (dependent variables), averaged across all participants with valid trials. *Error bars* indicate ± 1 standard error. Condition labels indicate the visual target position and direction cue*. Hor. Error* refers to horizontal (*X)* reaching error relative to the target

Participants of our example data collection (*N* = 5; two female, three male; mean age 31.2 years, range 22–38 years) were researchers or student assistants in our lab, including two of the authors (IS and HK). All were right-handed as determined using the Edinburgh Handedness Inventory (EHI; Oldfield, 1971) with a mean handedness score of 82.2 (range 11–100), gave written informed consent and received no compensation for their participation. The experiment was approved by the research ethics board at Justus Liebig University Giessen and was run in accordance with the Declaration of Helsinki (2008). In contrast to a real study, where the experimenter would generally ensure that a participant adhered to the task instructions, participants here were encouraged to also generate a variety of “mistakes”, such as starting their movement before being cued to do so or looking at the target despite being asked to fixate centrally. Example data for these “mistakes” are shown below (negative movement latency in Fig. 4 and horizontal eye movements in Fig. 6) and highlight typical sources of data error in continuous behavior recording.

The experiment was run on a Dell Workstation PC (using an Intel Xeon W2135 CPU at 3.7 GHz, 32 GB RAM, 2 GB NVidia Quadro P2200 GPU; Dell Inc., Round Rock, TX, USA). During the task, participants wore an HTC Vive Pro Eye VR HMD (resolution 1440 × 1600 pixels per eye, 90-Hz refresh rate; HTC Corp., Xindian, New Taipei, Taiwan) and held a Valve Index controller (Valve Corp., Bellevue, WA, USA) in their right hand, which was used to perform and record the reaching movements. The eye tracker was calibrated using its built-in calibration routine at the start of the experiment. Additionally, five validation targets were presented (central and at ± 5° in a cross formation, see also Fig. 2) to determine the accuracy of the eye-tracking calibration. Average calibration accuracy across targets ranged from 0.40° to 0.63° (mean, 0.51°), which is well in line with the manufacturer supplied range of 0.5–1.1° (HTC Corporation, 2021).

Each trial started with the presentation of a white fixation sphere (diameter 5 cm, distance to observer 50 cm), which participants had to fixate for 1 s to begin the trial (see also Fig. 3, bottom panel, for a visualization of the trial sequence). Upon fixation, the sphere changed color to indicate the reach direction (blue: towards the target, red: opposite of the target) and a white target cube additionally appeared for 1 s at the same size and distance as the fixation sphere, randomly positioned 30 cm left or right of the participant’s body midline. Participants were instructed to wait for the sphere and target cube to disappear, then reach to the correct target location while keeping their gaze at the remembered location of the fixation sphere. Reaching movements were therefore performed in an empty scene with only the floor visible to avoid any spatial biases. Trials ended when the right controller crossed a distance threshold of 45 cm along the depth (*Z*) axis, followed by a text instruction to start the next trial by button press.

The study design systematically manipulated the factors target position (left or right hemifield) and direction cue (pro or anti). Additionally, in any given trial the controller could either be visible, providing visual feedback of the reach movement, or hidden (this manipulation of controller visibility was included to illustrate how stimulus properties can be changed on a trial-by-trial basis in the code). Together, these factors yielded a 2 (target position) × 2 (direction cue) × 2 (visual feedback) design. Every combination was repeated ten times, leading to a total of 80 trials per participant that took around 5 min including setup and calibration. Because the experiment only serves as a case study to illustrate the code and recorded data, we here focus on the implementation and extraction of common experimental variables and chose not to include any statistical analysis of the data.

### Implementation

The sample experiment is implemented as a single script file (pro_anti_reach.py) that only depends on Python modules bundled with the default Vizard 6 installation. Initialization of SteamVR components closely follows the examples provided with Vizard. In the following section, we therefore focus on the experimental paradigm and refer the reader to the Vizard documentation and bundled tutorials for specific guidance about setting up Vizard for VR paradigms.

When first instantiating an Experiment object to hold trial and participant information, global configuration parameters are imported from a JSON file (config.json). These values can also be added and modified via the object’s config attribute using Python dictionary syntax at any time. Likewise, the basic experimental design is read from a comma-separated value (CSV) text file (trials.csv). This separation of study parameters and experiment code is usually good practice, as it allows to use the same code to rapidly pilot different stimulus parameters or run multiple sessions with different experimental conditions by providing a different trial file each time.^4^ The design is then multiplied by the number of repetitions specified in the config file and the resulting trial list (8 factor combinations × 10 repetitions = 80 trials) is randomized. After building the trial design, we construct a simple virtual scene containing a ground plane and geometric primitives as fixation and target stimuli (Fig. 3, top) using only resources bundled with Vizard. The fixation sphere and target cubes are added to an ObjectCollection to demonstrate the functionality to concisely show and hide different stimuli. Because we are interested in recording eye movements relative to the target and fixation objects, an invisible, fronto-parallel plane is placed at target distance from the observer before adding the SampleRecorder component to the experiment. When using an eye tracker, the sample recording task automatically performs an intersection test between the observer’s gaze direction vector and objects in the scene. This test is run on each display refresh, and the resulting 3D gaze position and currently fixated object can be stored in the log file. To ensure that we only record 3D gaze points within the target plane, we here disable intersection tests for all other stimulus objects. To also record the participant’s hand movements, we add the controller node to the sample recording task. Finally, we implement a virtual proximity sensor using Vizard’s vizproximity module that will later be used to detect the participant’s reach movement and end the trial.

As is typical for Vizard scripts, the main experiment functionality is contained in a task function (implemented as a Python “generator”, meaning a function that contains at least one yield statement), which is passed to the Vizard scheduler to run. This enables interactive functionality such as the participant metadata UI while not interrupting the Vizard rendering loop, and is similar to the use of coroutines in Unity (Bebko & Troje, 2020; Brookes et al., 2019). At the beginning of the experiment, we collect standard participant metadata, calibrate and validate the eye tracker if present, and set the visual stimuli to always appear at the participant’s eye height. Individual trials are implemented using trial preparation (TrialSetup) and main trial (TrialTask) task functions in combination with the Experiment.run() method. Although the trials here are fairly simple and could be implemented in a single task function, we chose to include a trial preparation task to show how functionality can be divided for code readability. In preparation for each trial, all stimuli are hidden and controller visibility is set according to the current trial’s feedback parameter. The code then presents a message to the participant and waits for controller button press to continue into the main trial task. The main task implements the paradigm as described above, using general stimulus information from the experiment’s config as well as the params of the currently running trial to set up the behavioral conditions. Result data are written to the current trial object’s results structure. After all trials have been run, experimental data is saved to a JSON file containing all data stored in the current experiment as well as to individual tabular text files. This serves to illustrate both data formats in the example dataset provided. Note that as the text file format (e.g., one table row per trial for trial result data) is likely familiar to many researchers, our analysis code in the GitHub repository focuses on extracting specific data from the JSON format. In addition to the scenario described above, we also provide a standalone version of the same experiment (pro_anti_reach_standalone.py) that illustrates how the same result can be achieved by specifying information such as stimulus parameters and trial design directly in the Vizard script, without relying on external files.

### Results and example dataset

We used Python (version 3.7) for data processing. Data were imported from the JSON files created after each experimental session. Trials with a movement latency or movement duration (see below for definitions) above or below three standard deviations from the respective mean were removed as outliers. No other data processing or selection was performed in this example analysis.

As a first step, we calculated the rate of correct responses as the proportion of trials in which participants reached towards the correct hemifield based on target location and reach cue. Except for one trial of one participant, all responses fell into the correct hemifield, which is not surprising given the relatively low difficulty of the task. Movement onsets were then extracted from the sample data using a velocity criterion. Controller velocity in the *X*, *Y*, and *Z* axes was calculated by differentiating the recorded position data, and movement onset time was defined as the sample where positive *Z* velocity (away from the participant) exceeded a threshold value. Using the movement onset time, movement latency was then defined as the time between the go cue (disappearance of the fixation and target stimuli) and movement onset, and movement duration was defined as the time between movement onset until the controller first crossed the proximity sensor at 45-cm distance, ending the trial (see Fig. 4). Finally, horizontal endpoint error is defined as the absolute distance along the *X* direction between the point where the controller first reached the distance threshold and the current trial’s target position. Individual and group averages of all three measures are displayed in Fig. 4, split by each combination of target position and reach cue. One participant (#5) was asked to start their movements “early”, i.e., before the visual cue. Figure 4 clearly shows that this resulted in negative movement latencies, as well as unrealistically long movement durations due to the fact that the proximity sensor was crossed on their way back to the starting position. These results emphasize the importance of running pilot experiments before the actual data collection, which is greatly facilitated in our solution by separating the code and experimental design.

**Fig. 5:**
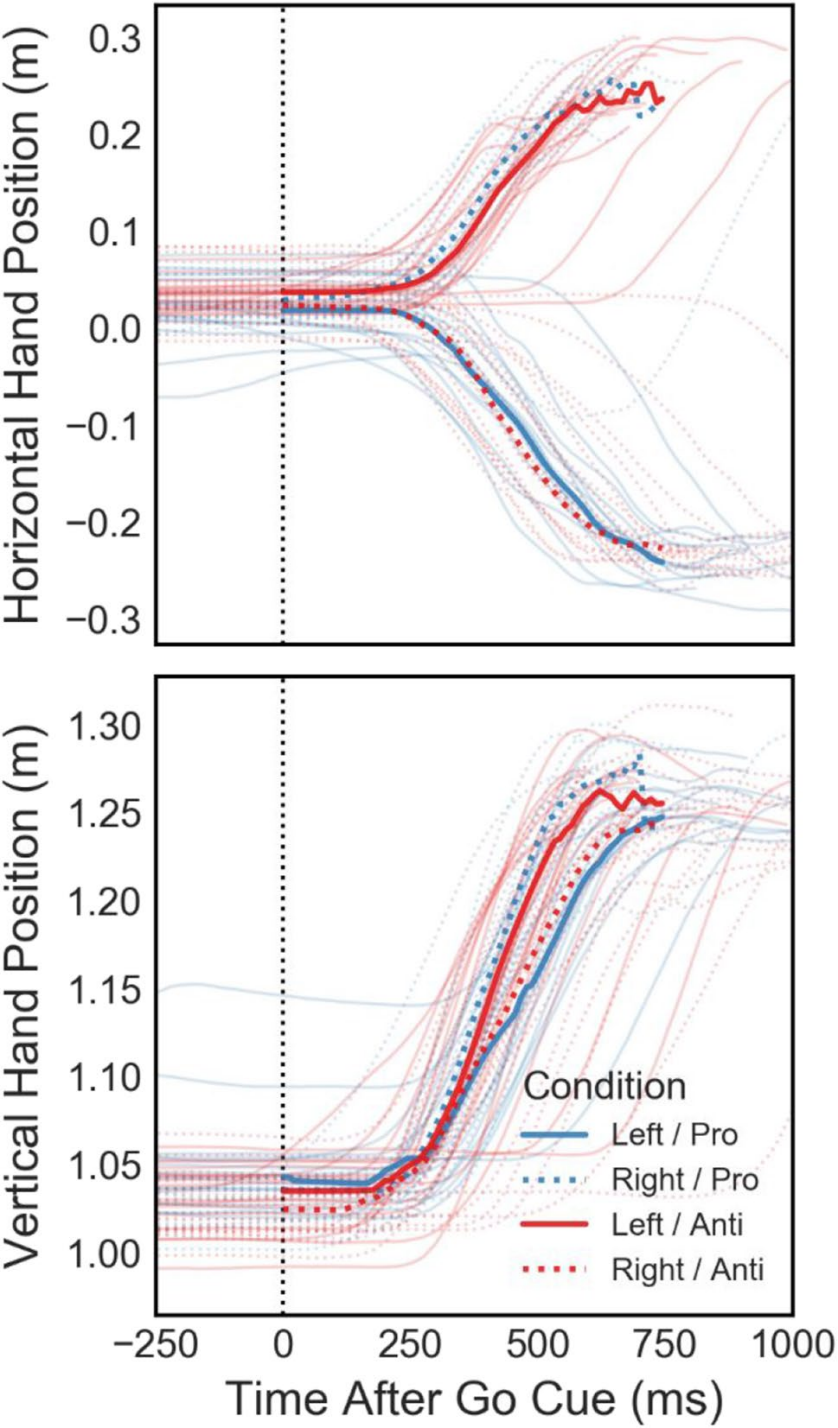
Example movement data for the individual conditions from all trials performed by one participant (#2). Panels show the right controller position over time, relative to the disappearance of target and fixation stimuli (go cue—*dotted vertical line*)

**Fig. 6:**
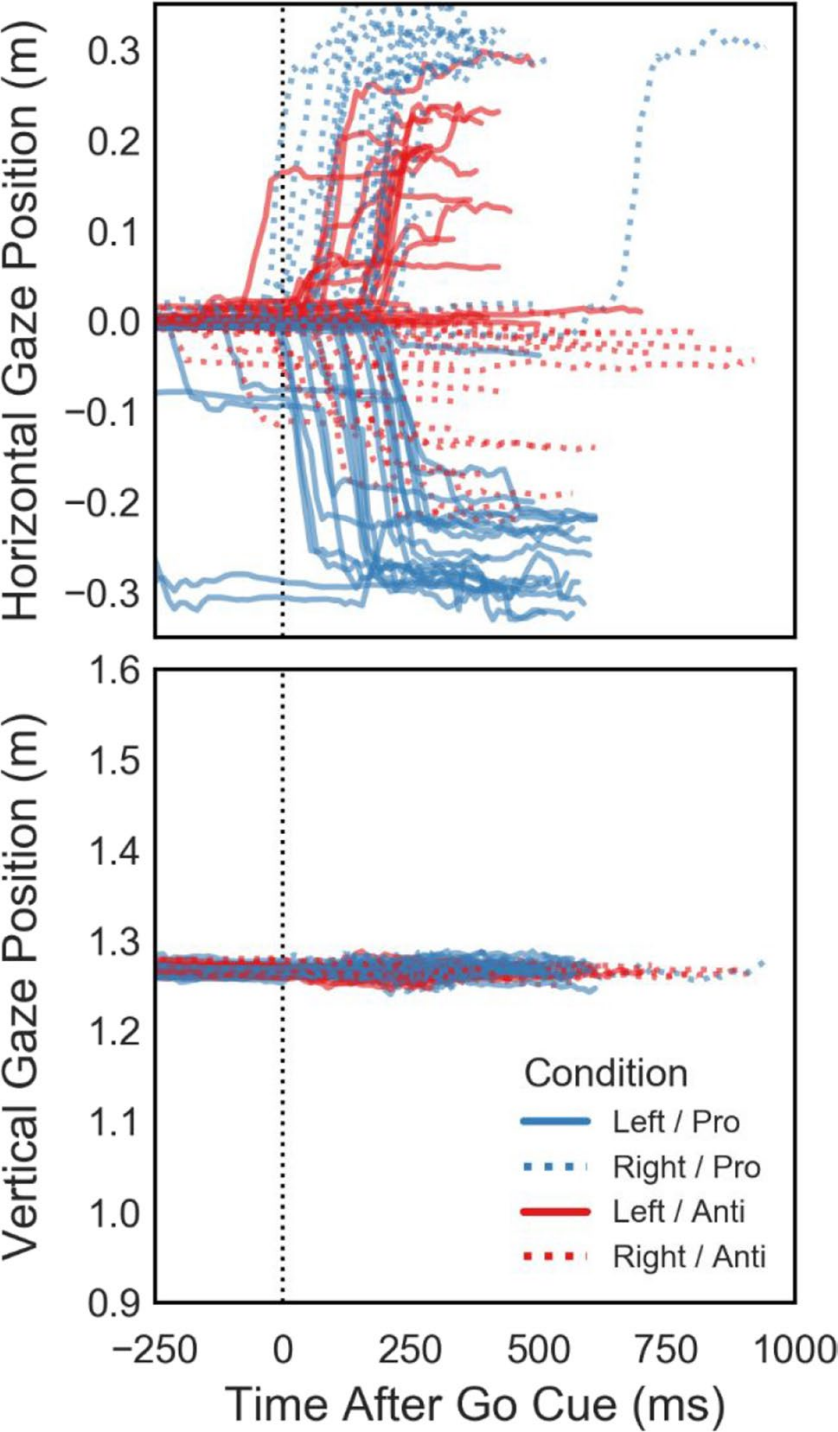
Example horizontal and vertical gaze position in the VR environment during individual trials in one participant (#1), plotted relative to the disappearance of target and fixation stimuli (go cue—*vertical dotted line*)

A VR experiment enables researchers to investigate continuous behavior in addition to individual trial results, in this case by recording controller movements at each display refresh. Movement data for one example participant (#2) is plotted in Fig. 5. Thin lines show movement trajectories in individual trials, split by target and reach cue combinations. Thick lines illustrate averaged trajectories per condition for the first 750 ms of movement after the go cue (dotted vertical line). While we did not perform any kinematic analysis here, the movement data is very systematic and shows the viability of controller position data recorded at a 90-Hz sampling rate in studying dynamic behavior. In addition to controller movement, we also recorded participants’ gaze position on a virtual fronto-parallel plane and found average eye tracking accuracy to be around 0.5° (Fig. 2). To exemplify how gaze data can be valuable even in fairly simple experiments, the horizontal and vertical gaze position in the target plane of another example participant (#1) are shown in Fig. 6. Despite the instruction to fixate centrally throughout each trial, this participant was asked to perform eye movements towards the target in the horizontal (*X*) direction on a large number of trials, but did not shift their gaze much in the vertical (*Y*) direction. In an experiment where the stimulus and/or reach goal location relative to the participant’s visual field is of importance, such as when studying lateralization using EEG, such eye tracking data can aid in detecting and removing invalid trials during analysis or even detect improper fixation during a trial, allowing the experiment to repeat the trial if necessary.

## Discussion

In the present manuscript, we have outlined the major features of our open-source software vexptoolbox for behavioral studies on the Vizard platform. To our knowledge, this is the first such framework for Vizard, complementing a wide range of software tools already available for Unity. A researcher’s choice in rendering engine can be influenced by different factors, such as their experience with the corresponding programming language and support for specific hardware components. Unity and Unreal often receive early support and official Software Development Kits (SDKs) from manufacturers of new consumer hardware, such as new head-mounted displays (HMDs), controllers etc., due to their large market share. At the same time, Vizard includes built-in support for a large range of research-focused hardware that can be complicated to integrate with Unity or Unreal, such as force-feedback devices, haptic displays, and physiological data acquisition tools. We provide our toolbox for Vizard as open-source to facilitate the creation of VR experiments for researchers working on this platform, help avoid frequent re-implementations of common functionality and make experiment code easier to understand and share.

Some of the design decisions we made when implementing the current version of our toolbox also come with possible limitations. One example is the decision to run the sample recording task locked to the display frame rate (typically 90 Hz) and leave the updating of sensor information to the Vizard runtime. A major advantage of this approach is that any sensor device that can supply 3D data to Vizard is automatically supported by our code and no device-specific software development kit (SDK) needs to be included. However, hardware such as eye trackers already support faster sampling rates (i.e., 120 Hz for the built-in eye tracker in the Vive Pro Eye HMD), meaning that the eye tracking data may be artificially reduced in fidelity and/or introduce temporal jitter (Andersson et al., 2010; Schuetz et al., 2020). The same is true for SteamVR controllers and other motion tracking systems. This can be problematic with paradigms that require high temporal accuracy, such as studies employing gaze-contingent stimuli, on-line movement velocity calculations, or highly accurate reaction times. Such timing problems could be solved by e.g. using an external synchronization device (Watson et al., 2019) or by recording data in a background process separate from the rendering engine (Wiesing et al., 2020). We chose not to take this approach here in the interest of compatibility and ease of use, but researchers should be aware of the limitations of display-synced data recording when planning a VR experiment.

During the preparation of this manuscript, WorldViz released a first-party extension to Vizard (“SightLab VR”), which is specifically geared towards creating eye tracking experiments and available as a separate purchase. While there may be some superficial similarities between our toolbox and this commercial extension, given that both are meant to create experimental studies and include eye tracking as a behavioral measure, the two packages pursue different goals. The commercial package promises a “simple yet powerful tool for setting up eye tracking experiments in VR” (WorldViz website) and thus appears focused on ease of use for a specific type of study, while our toolbox is meant to aid in implementing any behavioral paradigm using Python code and is not specifically geared towards eye tracking studies. It is also important to note that none of the authors of the presented manuscript or toolbox have licensed or used the SightLab extension. Based on the publicly available brochure, the two approaches target different problem spaces but can be complementary depending on the planned research project.

We plan to support and extend our toolbox in the future and encourage feedback and bug reports using the GitHub issue tracking features. Some features already planned for future releases include event detection for movement data (such as a detection of saccades and fixations for eye movements or inclusion of a velocity-based movement onset criterion as used in the example analysis) and a user interface for the experimenter to view the current trial parameters while the experiment is running. Nonetheless, we hope that our first release will already prove useful to other researchers in using virtual and mixed reality as a behavioral research method.

## Data Availability

Experiment code, anonymized data, and analysis code for the example study are available in a GitHub repository: https://github.com/ischtz/proantireach-vizard

## Footnotes

1 https://github.com/ischtz/proantireach-vizard

2 https://github.com/ischtz/vizard-experiment-toolbox

3 See Vizard documentation for details on Vizard’s task scheduling system.

4 Note: Calling *addTrialsFromCSV* without a file name will show a file dialog for easy selection.

## Code availability

The software toolbox is available at: https://github.com/ischtz/vizard-experiment-toolbox
